# ZnO Nanowire Networks as Photoanode Model Systems for Photoelectrochemical Applications

**DOI:** 10.3390/nano8090693

**Published:** 2018-09-06

**Authors:** Liana Movsesyan, Albert Wouter Maijenburg, Noel Goethals, Wilfried Sigle, Anne Spende, Florent Yang, Bernhard Kaiser, Wolfram Jaegermann, Sun-Young Park, Guido Mul, Christina Trautmann, Maria Eugenia Toimil-Molares

**Affiliations:** 1 Materials Research Department, GSI Helmholtz Centre for Heavy Ion Research, Planckstr. 1, 64291 Darmstadt, Germany; l.movsesyan@gsi.de (L.M.); wouter.maijenburg@chemie.uni-halle.de (A.W.M.); n.goethals@gsi.de (N.G.); a.spende@gsi.de (A.S.); C.Trautmann@gsi.de (C.T.); 2 Material- und Geowissenschaften, Technische Universität Darmstadt, Alarich-Weiss-Str. 2, 64287 Darmstadt, Germany; F.Yang@gsi.de (F.Y.); kaiser@surface.tu-darmstadt.de (B.K.); jaegermann@surface.tu-darmstadt.de (W.J.); 3 Stuttgart Centre for Electron Microscopy, MPI for Solid State Research, Heisenbergstr. 1, 70569 Stuttgart, Germany; w.sigle@fkf.mpg.de; 4 Photocatalytic Synthesis Group, MESA+ Institute for Nanotechnology, Faculty of Science and Technology, University of Twente, 7500 AE Enschede, The Netherlands; sypark100@gmail.com (S.-Y.P.); g.mul@utwente.nl (G.M.)

**Keywords:** etched ion-track membrane, electrodeposition, nanowire network, core-shell nanowires, ZnO, TiO_2_, photoelectrochemical applications, water splitting

## Abstract

In this work, the fabrication of zinc oxide (ZnO) nanowire networks is presented. By combining ion-track technology, electrochemical deposition, and atomic layer deposition, hierarchical and self-supporting three-dimensional (3D) networks of pure ZnO- and TiO_2_-coated ZnO nanowires were synthesized. Analysis by means of high-resolution transmission electron microscopy revealed a highly crystalline structure of the electrodeposited ZnO wires and the anatase phase of the TiO_2_ coating. In photoelectrochemical measurements, the ZnO and ZnO/TiO_2_ nanowire networks, used as anodes, generated higher photocurrents compared to those produced by their film counterparts. The ZnO/TiO_2_ nanowire network exhibited the highest photocurrents. However, the protection by the TiO_2_ coatings against chemical corrosion still needs improvement. The one-dimensionality of the nanowires and the large electrolyte-accessible area make these 3D networks promising photoelectrodes, due to the improved transport properties of photogenerated charge carriers and faster redox reactions at the surface. Moreover, they can find further applications in e.g., sensing, catalytical, and piezoelectric devices.

## 1. Introduction

In recent years, the fabrication and development of semiconductor three-dimensional, hierarchical nanowire assemblies has attracted increasing interest. The compact design, mechanical stability, and high surface area of 3D nanowire-based networks can be advantageous in enhancing the performance of gas sensors [1,2], batteries [3,4], and supercapacitors [5], as well as facilitating efficient light absorption and charge carrier transport in photoelectrodes [6,7,8,9,10,11,12,13,14] and optical elements [15,16]. Photoelectrodes can be applied, for example, in water splitting devices for the generation of H_2_ as renewable solar fuel. In 1972, Fujishima and Honda successfully demonstrated light-assisted water splitting using an n-type TiO_2_ photoanode connected with a platinum black electrode in a photoelectrochemical (PEC) cell for hydrogen production [17]. Since then, increasing the efficiency of both photon-to-current conversion and hydrogen generation has remained a major challenge for scientists. Improvements have been obtained by combining several materials in a tandem cell and adding co-catalysts to open the door for more efficient reduction and oxidation reactions, as well as designing different configurations of photoelectrochemical cells [18]. Besides efficiency, also cost, ease of fabrication, and robustness are also essential requirements that photoelectrodes need to fulfil.

In this work, we investigated the possible path of enhancing the efficiency of photoelectrochemical water splitting by using nanostructured materials [19]. In particular, nanowire-based photoelectrodes are of interest due to the properties arising from their one-dimensional geometrical orientation, which is expected to better facilitate charge carrier separation and transport to the surface of the electrode, compared to bulk materials [20]. Also, the reduced dimensions and large surface area of the photoelectrode/electrolyte interface may lead to shorter diffusion paths for the generated charge carriers, minimized recombination, and faster redox reactions. In addition, nanomaterials can be synthesized in the form of single-crystals, thus significantly decreasing carrier recombination such as at grain boundaries, and potentially also exhibiting increased optical absorption compared to their bulk counterparts [8].

Among the various methods currently available for synthesizing nanowires, electrochemical deposition in porous membranes is highly flexible, allowing the synthesis of a variety of materials including metals, semimetals, metal oxides, and semiconductors [21,22,23,24,25,26,27,28,29]. In particular, the electrodeposition of ZnO nanowires in polymer etched ion-track templates has been reported by several authors employing various electrolytes [30,31,32]. TiO_2_ nanowires have been also synthesized by applying a combination of sol-gel methods and polymer track-etched membranes [33]. The nanowires fabricated using this technique typically exhibit a high aspect ratio. The control over size, geometry, and surface area allows us to develop nanowire-based electrodes and to systematically investigate size-effects that influence processes such as electrical resistivity, wettability, surface reactivity, etc.

Here, we present the synthesis of three-dimensional (3D) networks of ZnO nanowires by electrodeposition inside the interconnected, etched ion-track channels of polycarbonate membranes. The presence of junctions between wires from different directions confer enhanced mechanical stability to the assemblies of high aspect ratio nanowires [27], thus providing self-supporting n-type ZnO nanostructured electrodes. The 3D nanowire networks were optimized in terms of integration density and wire diameter. To improve their chemical stability during measurements in aqueous media, the ZnO networks were coated with 20 nm TiO_2_ by atomic layer deposition (ALD). Pure ZnO and core/shell ZnO/TiO_2_ nanowire networks were employed as model systems to study the photoelectrochemical performance of such hierarchical nanowire structures, as compared to their film counterparts.

## 2. Materials and Methods 

**Figure 1:**
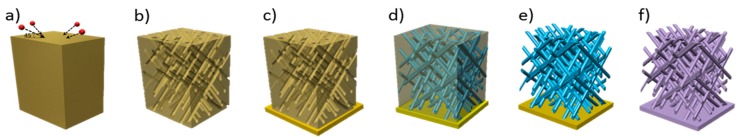
Schematic view of the different steps applied for nanowire synthesis: (**a**) Irradiation of polycarbonate films with heavy ions of GeV kinetic energy. The irradiation is performed in sequential steps from different angles of beam incidence; (**b**) chemical etching in 6M sodium hydroxide (NaOH) at 50 °C converts the ion-tracks into open nanochannels; (**c**) gold sputtering followed by electrochemical deposition provides a stable gold back-electrode; (**d**) electrodeposition of zinc oxide (ZnO) inside the membrane channels; (**e**) release of the ZnO networks by dissolution of the polymer template; (**f**) atomic layer deposition (ALD) of a titanium dioxide (TiO_2_) thin film conformally coating the wires and the gold substrate.

Series of ZnO and ZnO/TiO_2_ nanowire networks were synthesized by schematically following the steps presented in Figure 1.

Polymer templates with highly interconnected cylindrical nanochannels were fabricated by using the ion-track technology, which is based on the irradiation of MeV-GeV heavy ions creating ion-tracks that completely penetrate through the polymer foil. Chemical etching converts the ion-tracks into open nanochannels (Figure 1a,b). Subsequently, a working electrode is prepared on one side of the template (Figure 1c) and ZnO is electrodeposited inside the channels (Figure 1d). The polymer membrane is then dissolved in an organic solvent (Figure 1e). Finally, the resulting three-dimensional nanowire network is conformally coated by atomic layer deposition with a 20 nm layer of TiO_2_ (Figure 1f). To synthesize a ZnO/TiO_2_ nanowire network, approximately ~13 h are required—5 for the synthesis and 8 for the TiO_2_ ALD coating.

### 2.1. Membrane Fabrication

**Figure 2:**
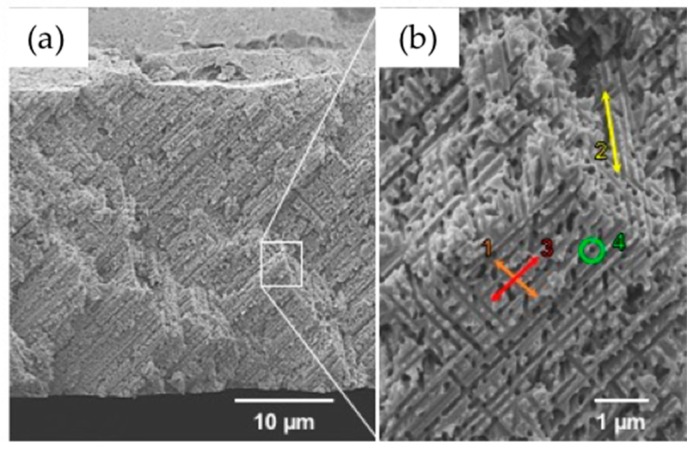
Scanning electron microscopy (SEM) images of (**a**) the cross-section of a representative template with high aspect ratio cylindrical interconnected channels, and (**b**) zoom-in indicating the four directions of the interconnected pores.

Polycarbonate foils (Makrofol N, Bayer AG) (30 µm thick, 30 mm diameter) were irradiated with ~2 GeV Au ions at the universal linear accelerator (UNILAC) of GSI in Darmstadt. Each ion passing through the foil creates, along its trajectory, a cylindrical damage zone known as an ion track. Intersecting tracks were obtained by irradiating the foils four times in consecutive steps, each time from a different direction under an angle of 45°, with respect to the surface normal of the polymer foil [24]. The foils were irradiated with a fluence of 4 × 5 × 10^8^, 4 × 10^9^ or 4 × 2 × 10^9^ ions/cm^2^ (2 × 10^9^, 4 × 10^9^ and 8 × 10^9^ ions/cm^2^ in total, respectively). Irradiation took place through a 5 mm diameter circular aperture (see details in Figure S1 of the Supplementary Information). To selectively remove the ion tracks, the irradiated foils were immersed in a 6 M sodium hydroxide (NaOH) solution at 50 °C. Under these conditions, the radial etching rate was ~11 nm/min. Prior to the etching, the foils were exposed to UV light (30 W, 312 nm T-30M, Vilber Lourmat, Eberhardzell, Germany) from both sides for 1 h to improve the etching of the tracks and the homogeneity of the diameter distribution [34]. To visualize the interconnected nanochannels and to analyze their geometry, membranes were additionally irradiated with high fluence heavy ions (10^10^ cm^−2^). The additional radiation damage combined with UV light exposure embrittled the samples and allowed us to break them in liquid nitrogen. This preparation process was applied to obtain the cross-section images shown in Figure 2.

### 2.2. ZnO Electrodeposition

The track-etched membranes with three-dimensional networks of channels subsequently served as templates for the electrochemical deposition of ZnO nanowire networks. First, one side of the template was sputtered with ~100 nm Au to make a conductive layer serving as an electrical contact to the polymer. The sputtered Au layer was then reinforced with a ~7 µm thick Au layer, which was electrodeposited in a two-electrode arrangement at *U* = −0.7 V using a commercial gold sulphite electrolyte (Gold-SF, 15 g Au L^−1^, METAKEM, Usingen, Germany) and an Au rod as anode. This layer served as a working electrode for the ZnO electrodeposition process, and later as a substrate for the nanowire networks.

ZnO was electrochemically deposited with a three-electrode setup, consisting of the Au back layer as a working electrode, a Pt spiral as a counter electrode, and Ag/AgCl (sat. KCl) (Sensortechnik Meinsberg GmbH, Waldheim, Germany) as a reference electrode. An aqueous electrolyte containing 0.1 M Zn(NO_3_)_2_·6H_2_O was used, and a constant potential ranging between *U* = −1 V and *U* = −1.4 V vs. Ag/AgCl at 60 °C was applied. Previously, electrodeposition of ZnO in etched ion-track membranes with parallel nanochannels was performed at *U* = −0.8 and −1 V vs. Ag/AgCl at 80 °C [35]. However, these depositions yielded very low filling rates in the interconnected pores. Cyclic voltammetry revealed a shift of the reduction peak to more negative potentials, namely, around *U* = −1.4 V versus Ag/AgCl. ZnO nanowire networks with a wire diameter of 150 nm were achieved by applying *U* = −1.4 V at 60 °C. Among a series of samples electrodeposited at 60, 70, and 80 °C, electrodeposition at *T* = 60 °C provided the most homogeneous fillings, which was most probably due to the reduced deposition rates. The process was monitored by recording the current-versus-time curve, and the deposition was stopped before the material reached the top of the channels [36]. Thus, the networks were grown until the channels were almost completely filled. As the material electrodeposited inside the channels replicate their shape, the deposition resulted in the growth of a 3D nanowire network. To release the network from the template, the polymer was carefully dissolved in a dichloromethane bath (CH_2_Cl_2_, Merck, Darmstadt, Germany).

In addition to the network structures, ZnO films were electrochemically deposited onto unirradiated PC films using the same electrolyte at 60 °C and applying cyclic voltammetry. First, metallic substrates were prepared by sputtering the polymer foils with ~100 nm Au, followed by electrodeposition of ~7 µm Au and ~3 µm Cu on top. Afterwards, the polymer was removed by immersing the sample for 2 h in acetone (Sigma Aldrich, Steinheim, Germany 99.99%), which enables easy detachment of the metallic layer from the polymer. Finally, the samples were cleaned with dichloromethane to remove any remaining polymer residues. The deposition of the ZnO films was carried out onto the sputtered Au layer by applying 10 cycles of cyclic voltammetry. The voltage was swept between 0 and −1.5 V versus Ag/AgCl with a scan rate of 10 mV/s. 

### 2.3. TiO_2_ Atomic Layer Deposition

Some of the prepared films and nanowire networks were conformally coated with ~20 nm TiO_2_ using a Picosun R200 ALD system. The main role of the TiO_2_ film was to protect ZnO from corrosion during photoelectrochemical measurements. The deposition was performed at 250 °C using titanium tetraisopropoxide (TTIP) and Millipore water as precursors, and nitrogen as carrier gas. Exposure times for TTIP and water were 1.6 and 0.1 s, respectively, for the 250 °C process. A few samples were coated with TiO_2_ at 110 °C, and used only for TEM analysis. In this case, the exposure times were 5.2 s and 0.4 s. The N_2_ purging times were 8 s and 50 s for 250 °C and 110 °C, respectively.

### 2.4. Photoelectrochemical Measurements

ZnO and ZnO/TiO_2_ nanowire network structures, as well as their film counterparts, were applied as photoanodes for the photoelectrochemical measurements. The setup available at the University of Twente consisted of an Arc lamp source with an Xe lamp (Newport 66902, 50–500 W, Irvine, CA, USA) calibrated at AM 1.5 (1 sun), a photoelectrochemical cell (PECC-2, ZAHNER-elektrik GmbH & Co. KG, Kansas City, MO, USA) for three-electrode measurements, and a potentiostat (VERSASTAT4, Princeton applied research, AMETEK, Inc., Berwyn, PA, USA). For the three-electrode setup a Ag/AgCl reference electrode was used, where the built-in Pt wire of the PECC-2 cell was the counter electrode, and the nanowire networks and films served as working electrodes. The samples were fixed with a special O-ring, with a pre-defined area of 0.196 cm^2^ being exposed to the light source (photoactive area). To turn the light on and off, a shutter was used. The measurements were performed in a 0.1 M K_2_SO_4_ (Sigma Aldrich) aqueous solution (pH 5.6). The position of the cell was adjusted with a calibrated Si solar cell (Newport Oriel, P/N 91150V, Irvine, CA, USA) to obtain an illumination of 1 sun. Linear sweep voltammetry (LSV) measurements were sequentially performed in the dark and under illumination by sweeping the voltage from 0.06 to 1.6 V_RHE_ (−0.55 V to 1 V_Ag/AgCl_) with scan rate 10 mV/s. Further chronoamperometric measurements were carried out for up to 3 h under chopped illumination (30 s light off/30 s light on), applying 0.62 V vs. Ag/AgCl. For ease of comparison with the values reported in literature, all potentials related to the PEC measurements were converted to the reversible hydrogen electrode (RHE), according to the Nernst equation:

(1) $$E_{RHE}=E_{Ag/AgCl}+0.059\;pH+E_{Ag/AgCl}^{o}$$

where $E_{RHE}$ is the converted potential versus RHE, $E_{Ag/AgCl}$ is the potential applied experimentally versus the Ag/AgCl (sat. KCl) reference electrode used, and $E_{Ag/AgCl}^{o}$ = 0.197 V is the standard potential of an Ag/AgCl reference electrode at 25 °C.

One set of linear sweep voltammetry (LSV) measurements (Figure 9) was performed using a different set-up available at TU-Darmstadt (Group of Prof. W. Jaegermann). In this case, the potentiostate was a GAMRY Interface 1000, and the pH value of the 0.1 M K_2_SO_4_ electrolyte was 5.6. The other parts, such as the photoelectrochemical cell, electrodes, and solar simulator, were comparable to the set-up employed at the University of Twente, as described above.

### 2.5. Sample Characterization

The morphology of both films and nanowire networks were characterized before and after ALD coating with a high-resolution scanning electron microscope (HR-SEM, JEOL JSM-7401F, JEOL, Akishima, Tokyo, Japan). In addition, parts of the wires of the networks were transferred onto transmission electron microscopy (TEM) grids. Electron energy loss spectroscopy (EELS) was used for chemical composition analysis. High-resolution transmission electron microscopy (HRTEM, JEOL-ARM200CF, JEOL, Akishima, Tokyo, Japan) and selected area electron diffraction (SAED) were used to analyze the crystallinity of these wires. The crystallographic properties before and after the PEC measurements were analyzed by means of X-ray diffraction (XRD) using a D2 PHASER diffractometer (Bruker, Billerica, MA, US) (Cu Kα, 30 kV, 10 mA).

## 3. Results

### 3.1. Network Analysis

Figure 2 shows representative SEM images of the cross-section of a network template with a channel density of 4 × 5 × 10^8^ cm^−2^ and a channel diameter of ~150 nm at low (a) and high (b) magnification. It is evident that the templates are highly porous and consist of well-interconnected cylindrical channels.

A series of etched ion-track membranes with various channel diameters, and total channel densities varying between 2 × 10^9^ (i.e., 4 × 5 × 10^8^) and 8 10^9^ (i.e., 4 × 2 × 10^9^) cm^−2^ were employed as templates to investigate and optimize the electrodeposition of ZnO interconnected nanostructures. Because the diffusion and migration of Zn^2+^ ions in highly interconnected channels differ to that in parallel channels, more negative potentials need to be applied. We found that the most homogeneous growth occurred under potentiostatic conditions, applying *U* = −1.4 V vs. Ag/AgCl at 60 °C [35].

**Figure 3:**
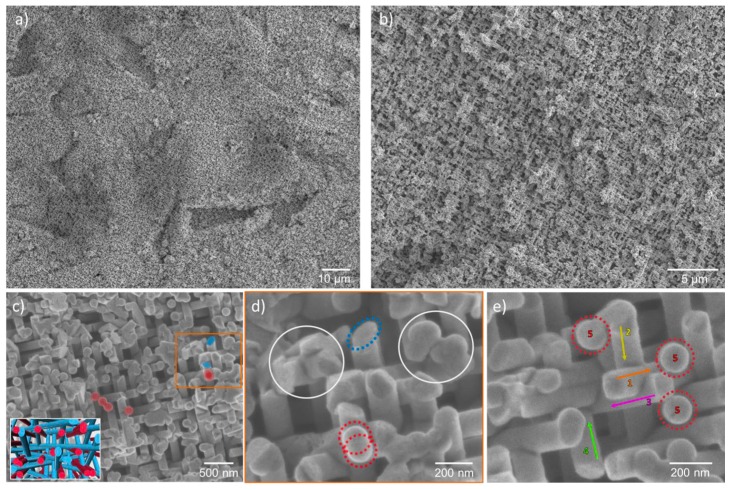
SEM images of a representative nanowire network with wire diameter ~150 nm and density ~5 × 10^9^ wires/cm^2^ at (**a**) low and (**b**,**c**) high magnification. The inset in (**c**) shows a 3D illustration of the top view of the network with wires tilted (blue) and perpendicular (red) with respect to the substrate; (**d**,**e**) zoomed-in SEM images of the network showing intersections and wire orientations.

After dissolution of the polymer, morphology and mechanical integrity of the nanowire networks were analyzed by high-resolution SEM (HRSEM). Figure 3 shows the SEM images of a representative network with 150 nm diameter wires along 5 directions (number density of wires ~5 × 10^9^ cm^−2^). The homogeneous growth of the network over the whole sample is visualized in Figure 3a over a ~100 × 120 µm area. Figure 3b,c show zoomed-in SEM images. In the inset, tilted and vertical wires are marked in blue and red, respectively. A higher magnification SEM image of the area marked in orange is shown in Figure 3d, which displays junctions between multiple wires. Regions marked in white illustrate that several wires interconnect at the same position. The advantage of this network structure is that the overlap of wires is mainly limited to junctions, keeping the one-dimensionality of the structure. Figure 3e shows the five different orientations. The total density of the resulting ZnO material can be adjusted by varying the nanowire density and/or the diameter. SEM images of nanowire networks with the same density of wires, namely 4 × 5 × 10^8^ cm^−2^, and wire diameters of 210, 150, and 90 nm are shown in the Supplementary Information (Figure S2). 

**Figure 4:**
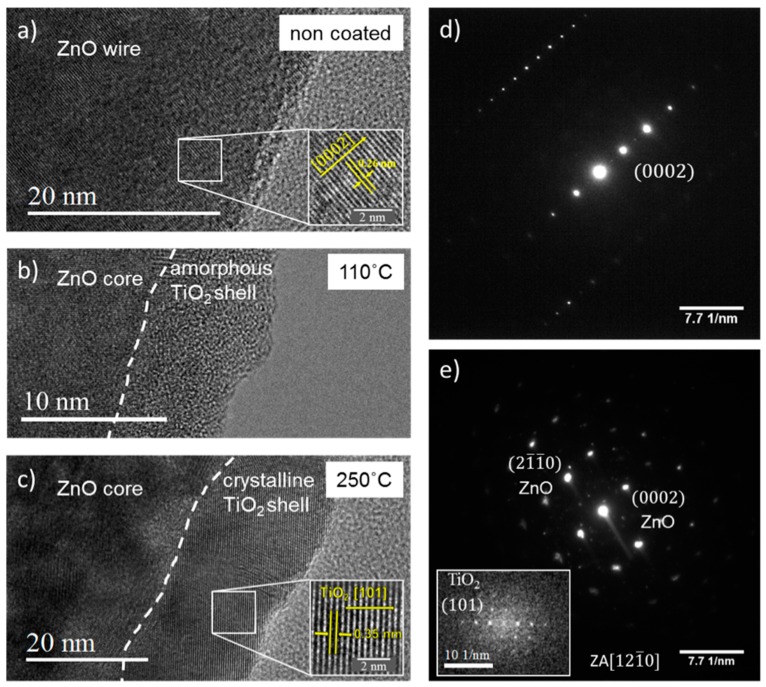
High resolution transmission electron microscopy (HRTEM) images of (**a**) ZnO and (**b**,**c**) ZnO/TiO_2_ nanowires. The TiO_2_ layer was synthesized by ALD at (**b**) 110°C and (**c**) 250 °C. The insets show high magnification images with measured *d*-spacings of 0.26 nm (**a**) and 0.35 nm (**c**) corresponding to the crystallographic planes of ZnO (0002) and anatase TiO_2_ (101), respectively. SAED patterns in (**d**,**e**) correspond to the ZnO and ZnO/TiO_2_ nanowires shown in (**a**,**c**), respectively. The inset in (**e**) shows the Fourier transform image of the crystalline TiO_2_ shell deposited at 250 °C.

To improve the chemical stability of the ZnO nanowire networks in aqueous solutions, they were coated with a thin layer of TiO_2_ using ALD. Figure 4 shows HRTEM images of the segments of (a) bare ZnO, (b) ZnO/TiO_2_ coated at 110 °C, and (c) ZnO/TiO_2_ coated at 250 °C. The HR-TEM image of the bare ZnO wire (Figure 4a) reveals an ordered set of planes. The measured distance between the displayed planes was 0.26 nm, as expected, which corresponds to (0002) planes of ZnO with a hexagonal wurtzite structure. The TiO_2_ layer deposited at 110 °C is amorphous (Figure 4b) while at 250 °C the anatase phase is formed (Figure 4c). The inset in Figure 4c also reveals an ordered set of planes corresponding to the TiO_2_ anatase phase (interplanar distance = 0.354 nm). The respective SAED patterns of uncoated (Figure 4a) and TiO_2_-coated (Figure 4c) wires are shown in Figure 4d,e.

**Figure 5:**
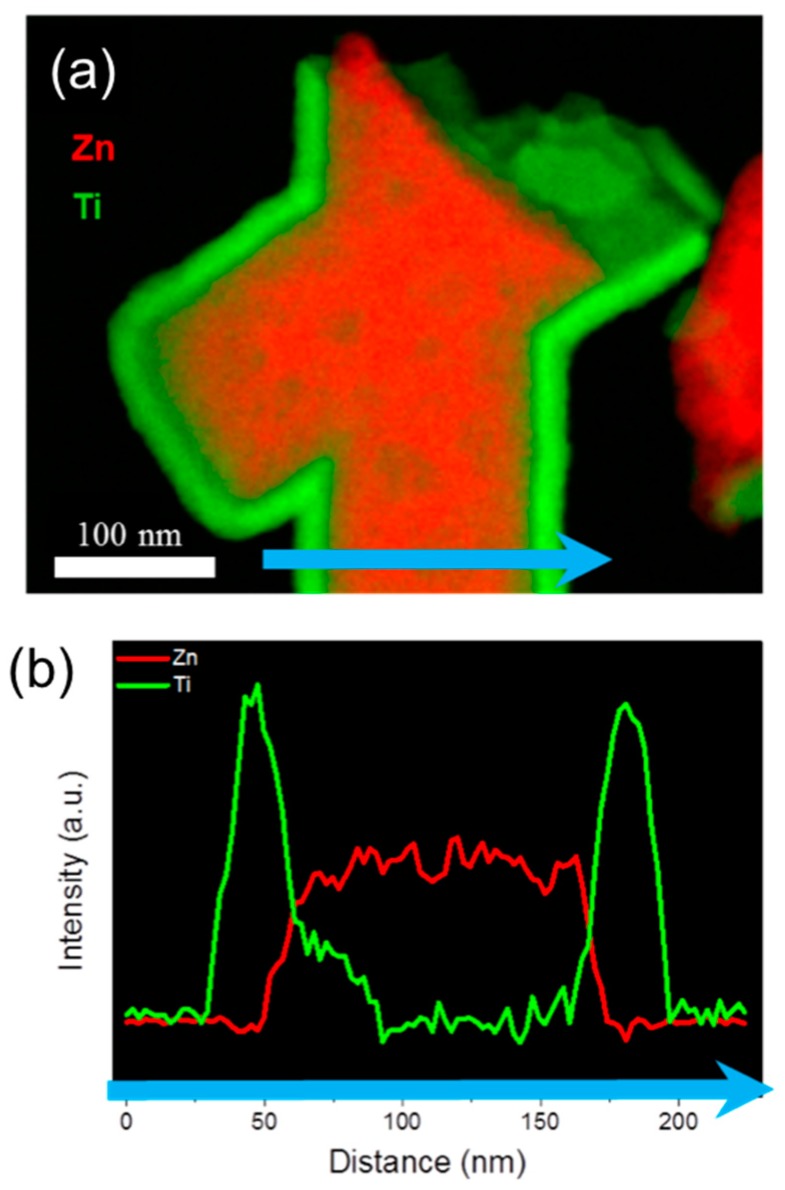
(**a**) 2D electron energy loss spectroscopy (EELS) elemental map of a junction of two wires, and (**b**) linescan across one nanowire, as marked in (**a**) by the blue arrow.

Figure 5a displays a superposition of the high-angle annular dark-field (HAADF) image from a junction of two ZnO/TiO_2_ nanowires, and the corresponding EELS elemental mapping. All the wires and intersections examined by SEM and TEM after ALD coating evidenced a consistent increase in wire diameter of ~40 nm. In particular, the pieces broken for the TEM studies can belong to either the top or the bottom part of the network, strongly indicating the homogeneous and conformal coating of the complete wire surfaces, including narrow geometries at intersections. A compositional line scan across the nanowire confirms the core-shell morphology (Figure 5b).

### 3.2. Photoelectrochemical Measurements

**Figure 6:**
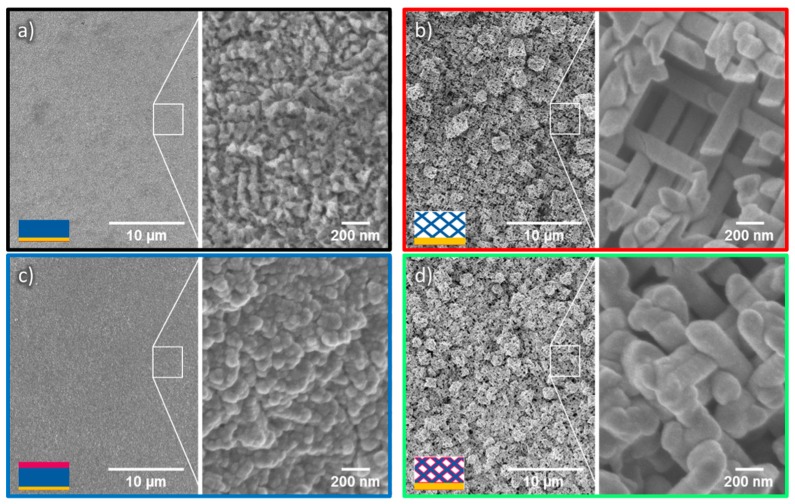
Low (left) and high (right) magnification SEM images, displaying (**a**) a ZnO film exhibiting a rough surface and spike-like features; (**b**) a ZnO nanowire network; (**c**), a ZnO film coated with 20 nm TiO_2_ and (**d**) a ZnO/TiO_2_ core-shell nanowire network. In the insets’ schemes, the colors present the different materials: gold (yellow), ZnO (blue), and TiO_2_ (pink).

For the photoelectrochemical measurements, several ZnO nanowire networks of identical characteristics—that is, of channel density 4 × 2 × 10^9^ cm^−2^, wire diameter 150 nm, and wire length ~35 µm—were prepared. ALD coatings were performed at 250 °C to obtain a 20 nm-thick, crystalline TiO_2_ layer. Corresponding planar films with thicknesses of 7–10 µm were used as reference samples. SEM images of the various networks and films are displayed in Figure 6. After TiO_2_ coating, both film and network samples appeared smoother. This morphology change is attributed to being exposed to 250 °C for 7 h during the ALD process. Analysis of the samples on the complete surface shows that the networks are homogeneous over the entire sample area of ~20 mm^2^.

**Figure 7:**
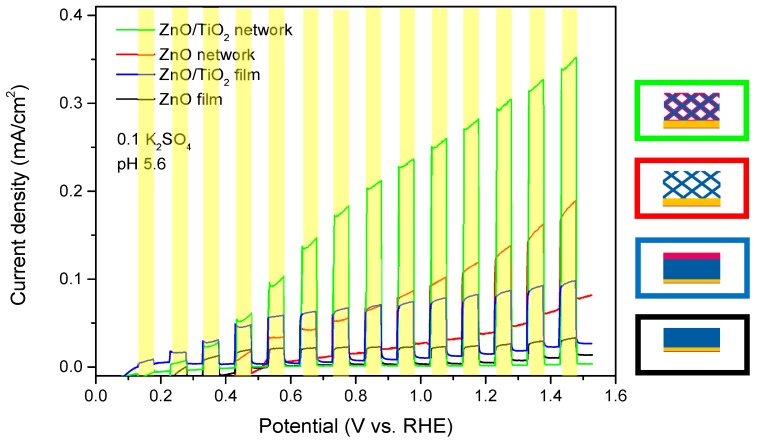
Linear sweep voltammograms of the bare ZnO, and TiO_2_-coated ZnO nanowire networks and films described in Figure 6. The nanowire density is 4 × 2 × 10^9^ cm^−2^ and the nanowire diameter is ~150 nm, in all cases. The 20 nm thick TiO_2_ coating layers were prepared by ALD at 250 °C. The LSVs were measured under chopped light illumination (light on areas marked in yellow) with a scan rate of 10 mV/s.

After their morphological characterization, the samples were mounted into the photoelectrochemical cell and LSVs were recorded for each of the four samples under chopped light conditions (Figure 7).

In the dark, the photocurrent density (~few hundreds of nA/cm^2^) is negligible for four samples for the complete potential range. The bare ZnO network exhibits a higher dark current, as expected. This is probably due to the direct access of electrolyte to the gold substrate at the bottom of the network, where adsorption of SO_4_^2−^ ions takes place [37]. Under illumination, the oxygen evolution reaction occurs at the photoanodes, and the resulting photocurrents are measured. The films exhibit low photocurrents (~0.02 mA/cm^2^ for ZnO and ~0.06 mA/cm^2^ for ZnO/TiO_2_, at 1.5 V vs. RHE) which remain constant or increase very slightly as a function of voltage. In turn, both 3D nanowire network samples (ZnO and ZnO/TiO_2_) display higher photocurrents which increased significantly with increasing potential, reaching ~0.1 and ~0.35 mA/cm^2^ at 1.5 V vs. RHE, respectively. In addition to its corrosion protection role, the TiO_2_ layer also increases the photocurrent for both the nanowire network and the film. This is attributed on the one hand to additional electron-hole pairs generated in the n-type TiO_2_, and, on the other hand, to the contribution of the ZnO–TiO_2_ interface to the charge separation, thereby reducing the probability of electron-hole recombination. These conclusions are supported by additional PEC measurements performed on TiO_2_ coated Au nanowire networks (Supplementary Information, Figure S3), as well as XPS measurements (Supplementary Information, Figure S4). Hu et al. discussed the stability and performance of devices coated with amorphous TiO_2_ and concluded that thicker (>100 nm) TiO_2_ layers provide better results in regard to electrical characteristics and stability [38]. Here, the thickness of the crystalline anatase TiO_2_ layer was limited to 20 nm due to the high density of the nanowire network.

**Figure 8:**
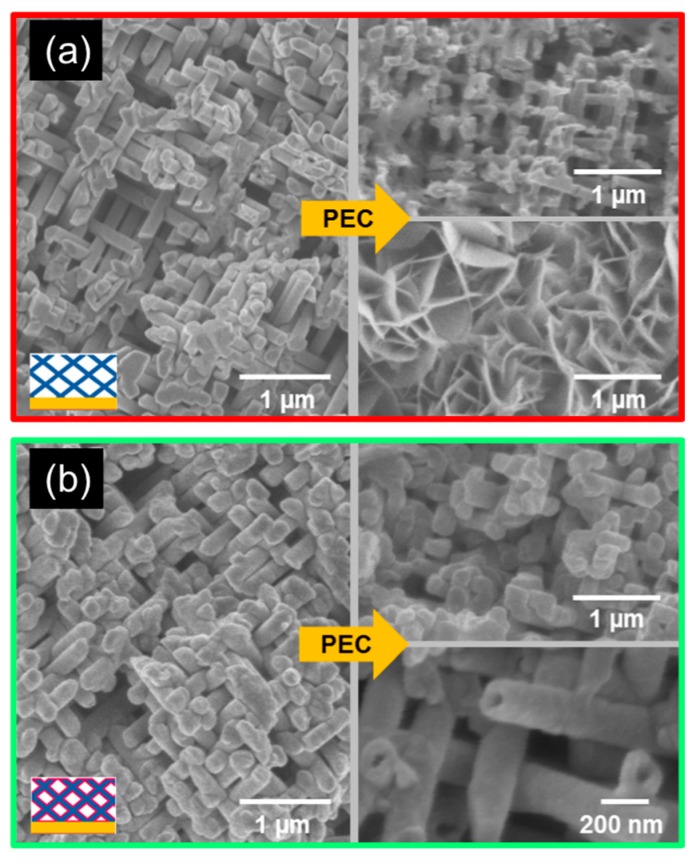
SEM images of (**a**) ZnO nanowire network and (**b**) ZnO/TiO_2_ nanowire network taken before (left) and after (right) PEC measurements. The insets schematically display the cross-section of each sample, indicating the distribution of the various materials—namely, gold (yellow), ZnO (blue), and TiO_2_ (pink).

To investigate the morphological stability of the nanowire network samples during the PEC measurements, the photoanodes were imaged again by SEM after the complete PEC characterization. The comparison of each sample before and after the PEC measurements is shown in Figure 8.

Figure 8a,b display the SEM top-view images of the ZnO and ZnO/TiO_2_ nanowire networks, after 1 h and 3 h of PEC measurements, respectively. The corrosion of the uncoated ZnO wires during the PEC measurements resulted in a decrease in wire diameter from ~150 nm to ~120 nm. Furthermore, in some areas, the morphology of the sample changed dramatically, exhibiting flakes with dimensions in the micrometer range, which we assume to be a result of recrystallization of the ZnO wires into the flake form due to the energy differences among different crystallographic facets [39].

It is well-known that ZnO, as many other candidate materials for water splitting, suffers from photocorrosion in aqueous solutions under ultraviolet illumination by hole-trapping on its surface [40]. ZnO can also decompose when the pH values of the solution are too low or too high, even without illumination [41]. The stability of bare ZnO nanowires during photoelectrochemical water splitting has been investigated by Liu et al. as a function of electrolyte anion and pH value [42]. 

Additional profilometry measurements (not shown) evidenced a decrease of the average height (thickness) of the nanowire network sample, from the initial 25 µm down to 10–15 µm. This is also consistent with the strong corrosion and recrystallization processes expected for bare ZnO samples. In contrast, the ZnO/TiO_2_ nanowire network did not undergo significant changes, demonstrating the protective role of the TiO_2_ layer. It is also worth mentioning that (in only on few small spots of the sample) empty TiO_2_ tubes were rarely observed (Figure 8b). This could indicate that the core of the ZnO/TiO_2_ nanowires in these small sample areas was dissolved, while the TiO_2_ shell remained stable. This should be investigated in more detail in future experiments. In this case, the average height of the ZnO/TiO_2_ network after PEC measurements remained ~25 µm, thus confirming a much higher stability of the TiO_2_-coated nanowire network.

**Figure 9:**
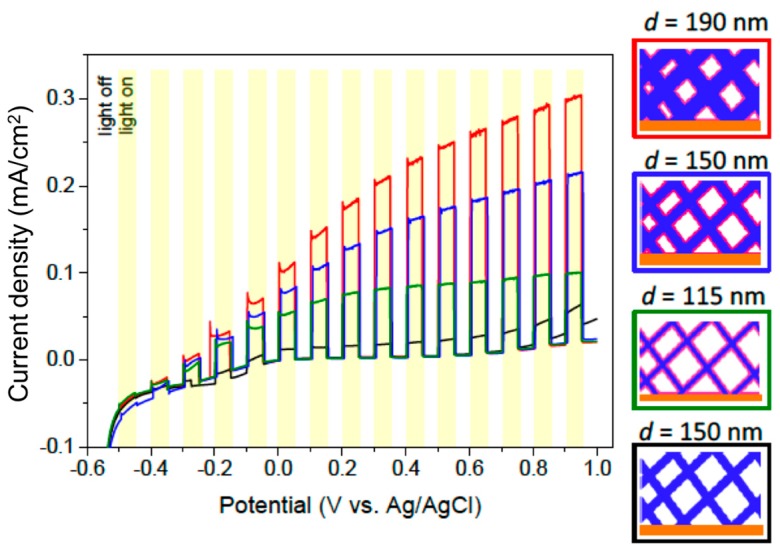
Linear sweep voltammograms measured under chopped light illumination for ZnO (black) and ZnO/TiO_2_ nanowire networks with nanowire density 5 × 5 × 10^8^ cm^−2^ and wire diameters 115 nm (green), 150 nm (blue), and 190 nm (red). The 4 nm TiO_2_ coating layer was prepared by ALD at 250 °C. Scan rate was 10 mV/s.

Figure 9 shows the LSVs recorded for the networks of ZnO nanowires with diameters of 115, 150, and 190 nm, all ALD coated with a 4 nm-thick TiO_2_ layer. The density of the nanowires was 5 × 5 × 10^8^ ions/cm^2^, the nanowires being assembled from 5 directions (see Supplementary Information, Figure S2). ZnO was electrodeposited in all membranes under the same conditions, using the same electrolyte and applying *U* = −1 V vs. Ag/AgCl at 60 °C. After dissolution of the polymer membrane, the nanowires were coated with 4 nm TiO_2_. A network with 150 nm-wide wires remained uncoated. The LSVs were recorded under chopped illumination conditions with a scan rate of 10 mV/s using a 0.1 M K_2_SO_4_ (pH = 5.6) solution.

At a given applied potential, the larger the diameter, the higher the generated photocurrent. This can be explained by the variation in the amount of photoactive ZnO material in the different samples. In addition, the variation of photocurrent as a function of potential increases with increasing nanowire diameter. To elucidate the influence of other effects, such as the wire diameter, compared to the carrier mean free path in such complex nanostructured systems seems challenging. In any case, all three ZnO/TiO_2_ samples exhibited photocurrent values larger than the corresponding films (see Figure 7). The TiO_2_ coating led also to larger photocurrents than the uncoated ZnO network.

Please note that ZnO has been used as a model system to investigate the influence of sample geometry on PEC performance. Due to the wide band-gap of ZnO, these networks absorb only UV photons, meaning higher photocurrent values are expected for the nanowire networks of other materials. Also, it is important to emphasize that the samples were measured as-grown, that is, without any post-annealing treatments, optimized ALD coatings, catalysts, or specific surface treatments. Consequently, significantly higher PEC values are expected after optimization of the samples.

## 4. Conclusions

In conclusion, three-dimensional nanowire networks of ZnO have been fabricated by electrodeposition in etched ion-track membranes and have been used as photoanodes in photoelectrochemical cells. Their geometrical parameters, such as wire density, wire diameter, and geometrical arrangement, can be easily adjusted by the different steps of the fabrication process. The nanowire networks released from the polymer template can be conformally coated by ALD. The resulting ZnO and ZnO/TiO_2_ nanowire networks exhibit high surface areas and increased light-induced photocurrents compared to their film counterparts. PEC characterization of these ZnO/TiO_2_ nanostructured model systems demonstrate that the 3D nanowire network geometry is very promising for the synthesis of electrodes for e.g., as water splitting. Finally, their mechanical stability also encourages their application for e.g., gas sensing, piezoelectrics, flexible electronics, etc. 

## Supplementary Materials

The following are available online at http://www.mdpi.com/2079-4991/8/9/693/s1, Figure S1: Irradiation through mask to limit the electrodeposition area during growth of interconnected nanowires, Figure S2: Nanowire network with interconnected wires from 5 directions, Figure S3: Photoelectrochemical properties of the TiO_2_ layer, Figure S4: ZnO/TiO_2_ band structure.
