# Supplementary material for: ZnO Nanowire Networks as Photoanode Model Systems for Photoelectrochemical Applications
Source: Nanomaterials (Basel). 2018 Sep 6;8(9):693. doi: 10.3390/nano8090693 (PMC6164027; doi:10.3390/nano8090693)
Supplement: Supplementary file 1 [file nanomaterials-08-00693-s001.pdf]

ZnO Nanowire Networks as Photoanode Model Systems for Photoelectrochemical Applications

Liana Movsesyan[1,2], Albert Wouter Maijenburg[1], Noel Goethals[1], Wilfried Sigle[3], Anne Spende[1,2], Florent Yang[2], Bernhard Kaiser[2], Wolfram Jaegermann[2], Sun-Young Park[4], Guido Mul[4], Christina Trautmann[1,2], Maria Eugenia Toimil-Molares*[1]

1 Materials Research Department, GSI Helmholtz Centre for Heavy Ion Research, Planckstr. 1, 64291 Darmstadt, Germany
2 Material- und Geowissenschaften, Technische Universität Darmstadt, Alarich-Weiss-Str. 2, 64287 Darmstadt, Germany
3 Stuttgart Centre for Electron Microscopy, MPI for Solid State Research, Heisenbergstraße 1, 70569 Stuttgart, Germany
4 Photocatalytic Synthesis Group, MESA+ Institute for Nanotechnology, Faculty of Science and Technology, University of Twente, 7500 AE Enschede, Netherlands.
* Correspondence: m.e.toimilmolares@gsi.de; Tel.: +49-6159-71-1807

SUPPLEMENTARY INFORMATION

1. Irradiation through a mask to limit the electrodeposition area during growth of interconnected nanowires.

The deposition area was limited by using a mask (diameter 5 mm) during irradiation (Fig. 1a). In this way, the electrodeposition area for the growth of the 3D network was smaller than the diameter of the cell compartment and that of the back-electrode, yielding a more reproducible and homogeneous growth of the 3D ZnO nanowire networks. When no mask is used during irradiation, the electrolyte leaks through the etched pores towards the Cu contact rings (Fig. 2b), causing more inhomogeneous growth and/or side effects.

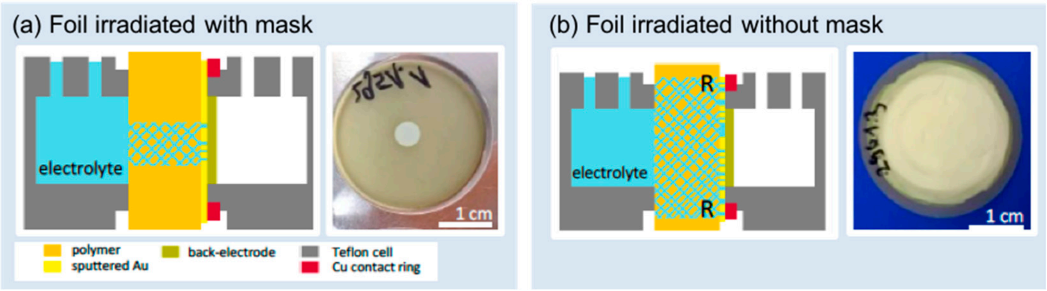

Figure S1. Schematics of the electrodeposition cell and the corresponding etched membranes irradiated a) with and b) without a mask. Without the mask, the electrolyte can leak through the pores in the areas marked with R. Corresponding photographs of polycarbonate-etched ion-track membranes irradiated with and without a 5 mm mask are also shown.

2. Nanowire networks with various wire diameters.

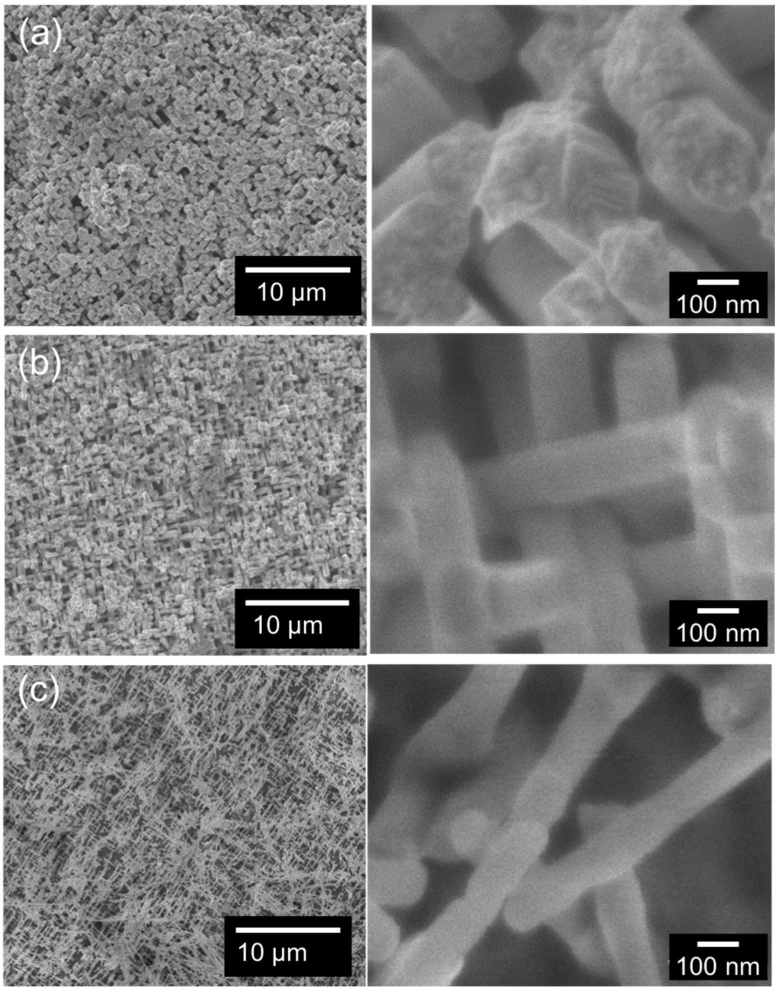

Figure S2. Scanning electron microscopy (SEM) images of nanowire networks with a wire density of $4 \times 5 \cdot 10^8$ cm^{-2} and average wire diameter of (a) 210, (b) 150, and (c) 90 nm. The insets show higher magnification images of a few interconnected wires.

A series of ZnO nanowire networks with the same density of wires ($4 \times 5 \cdot 10^8$ cm^{-2}) and decreasing nanowire diameters, namely (a) 210, (b) 150, and (c) 90 nm, were synthesized. These help to visualize how the total density of the resulting ZnO material can be adjusted. The networks with 210 and 150 nm nanowire diameter (i.e. Fig. S2(a) and S2(b)) are mechanically more stable than those with 90 nm diameter wires (see broken wires in Fig. S2(c)), and resemble a porous ZnO film. The wires are well-interconnected, and the interwire spacing increases with decreasing wire diameter. The thinner the nanowires, the larger the spacing between them (Fig. S2(c)). However, in this case, in some areas which are similar to the one displayed in the image, misaligned nanostructures are found.

3. Photoelectrochemical properties of the TiO_2 layer.

Figure S3 shows the schematic representations of the ZnO, Au/TiO_2 and ZnO/TiO_2 films (a-c) and nanowires network (d-f) photoanodes. The ZnO films have a thickness of 7-10 µm. The networks consist of interconnected wires with a length of ~30 µm and diameter of ~150 nm. The thickness of the TiO_2 layers is 20 nm for all coated samples.

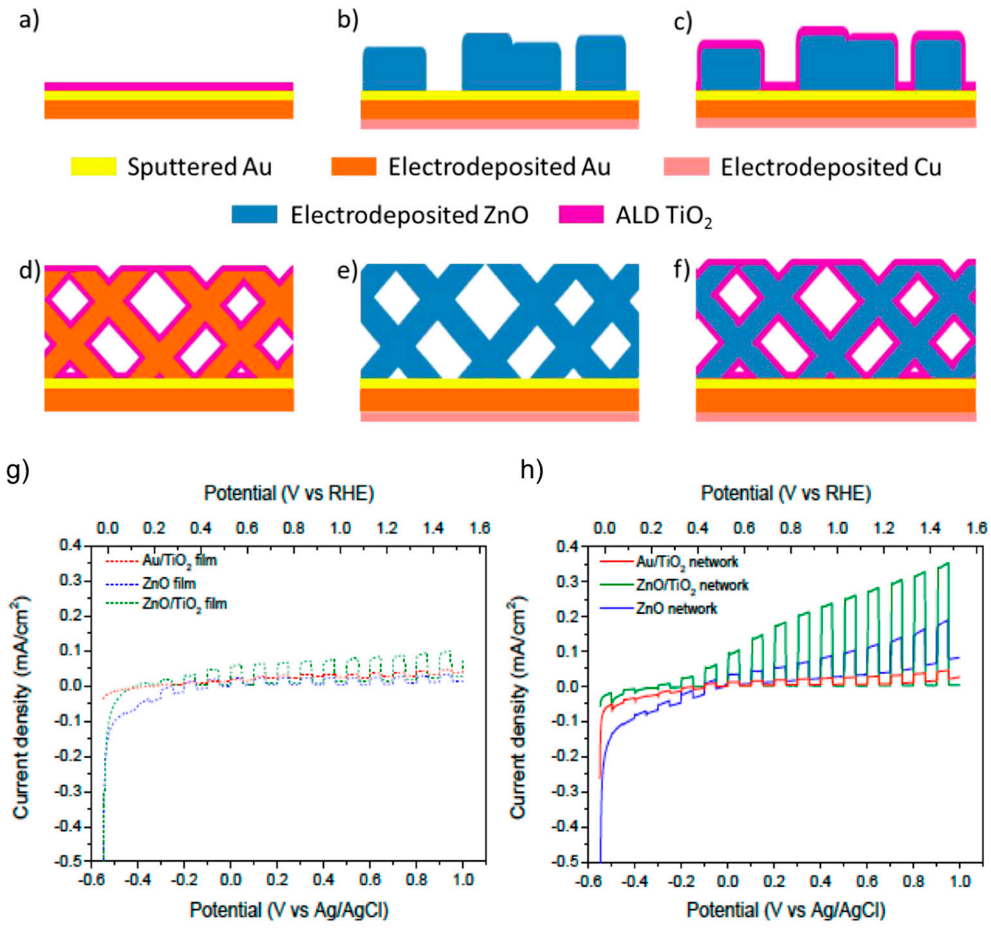

Figure S3. Schematic representation of (a-c) films and (d-f) nanowire networks of Au/TiO_2 (a,d), Au/ZnO (b,e), and Au/ZnO/TiO_2 (c,f). Linear sweep voltammetries recorded with 5 mV/s in 0.1M K_2SO_4 (pH 5.6) for (g) films and (h) nanowire networks of uncoated ZnO (blue), ZnO coated with 20 nm TiO_2 (green), and Au coated with 20 nm TiO_2 (red) are shown. The curves show the same trend in the photocurrent density, being the lowest for the only TiO_2-based, and the highest for the ZnO/TiO_2 photoanodes.

For the PEC measurements, the light source was an AM 1.5 simulated light corresponding to 1 sun. The electrolyte was a 0.1 M K_2SO_4 solution (pH 5.6). The dimensions of the uncoated (blue) and TiO_2-coated (green) ZnO films and networks are identical, meaning the relative increase in photocurrent can be attributed to the TiO_2 coating itself. In addition, this relative increase is significantly larger than the photocurrents produced just by the ZnO networks or by the TiO_2 coatings with same morphology and dimensions (red), which could indicate a contribution to the photocurrent by the ZnO–TiO_2 interface. However, a possible increase of the photocurrent due to the annealing of the ZnO nanowires during the ALD coating process at 250 °C should also be considered.

4. ZnO/TiO_2 band structure

The ZnO/TiO_2 interface can facilitate the charge separation due to a favorable band alignment for electrons and holes. X-ray photoelectron spectroscopy (XPS) (available in the group of Prof. W. Jaegermann at the Technische Universität Darmstadt) and UV/VIS spectroscopy were employed to further characterize the sample's materials. Figure S4 shows the valence band spectra of a ZnO nanowire network (a) and a 20 nm-thick TiO_2 ALD film (c), showing the valence band maximum (VBM) at 3.15 and 3.2 eV for ZnO and TiO_2, respectively. The corresponding secondary electron cutoffs are shown in (b) and (d), the work functions (WF) being 4.35 eV for ZnO and 4.05 eV for TiO_2. The WF of the Au back electrode is 5.1 eV. UV/VIS measurements provided optical band gap values of 3.28 and 3.3. eV, respectively. Based on these XPS and UV/VIS data, Fig. S4 (e) schematically shows the conduction band minimum and the valence band maximum of ZnO (green) and TiO_2 (violet). Under illumination, the electrons in the conduction band (CB) of TiO_2 would tend to transfer to the CB of ZnO and, correspondingly, the holes would flow from the valence band (VB) of ZnO to the VB of TiO_2. This would favor an efficient separation of photogenerated charge carriers by reducing the electron/hole recombination probability. Similar explanations were previously considered by Fan et al. [1] and Hernandez et al. [2].

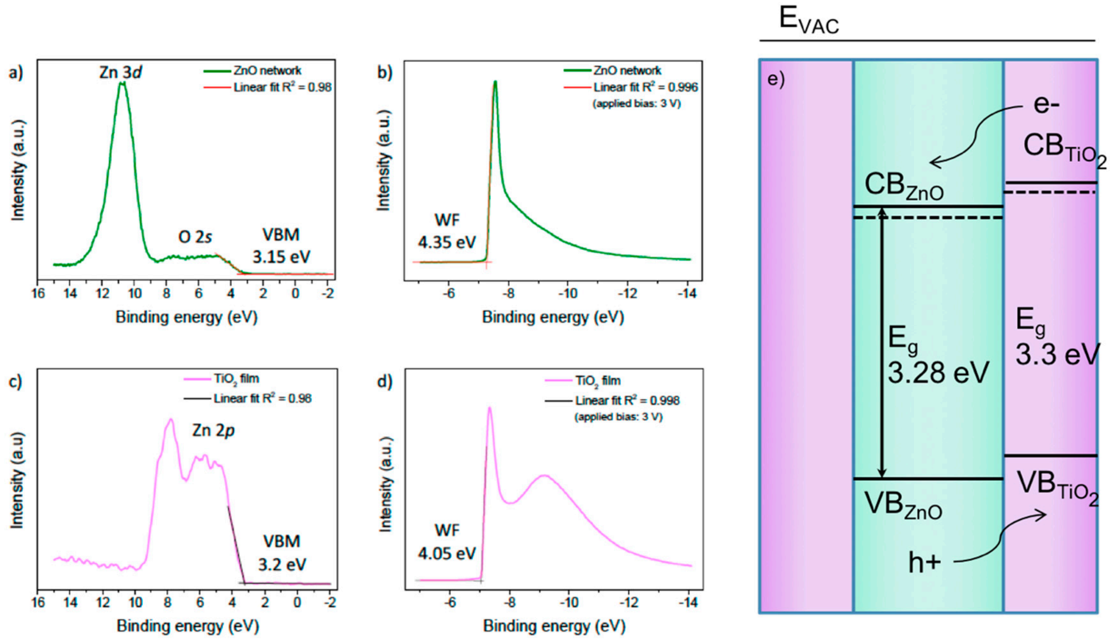

Figure S4. Valence band spectra of a ZnO nanowire network (a) and a TiO_2 film (c) showing the valence band maximum (VBM) at 3.15 and 3.2 eV for ZnO and TiO_2, respectively, as well as their secondary electron cutoff (b) and (d) from which the respective work functions (WF) of the ZnO and TiO_2 samples were found to be 4.35 and 4.05 eV.

The XPS measurements were performed at the DAISY-FUN (Darmstadt's Integrated System for Fundamental research) of the Surface Science group of Prof. Dr. Wolfram Jaegermann. The spectra were recorded by using a PHOIBOS 225 (Specs GmbH) analyzer and a monochromatic Al Kα radiation with an excitation energy of 1486.74 eV. Before each measurement, the system was calibrated with a high-purity Ag sample (freshly sputter-cleaned under Ar atmosphere) to calibrate the Fermi level. For determination of the work function, a negative bias of 3 V was applied between the analyzer and the sample for distinguishing the signal from the sample.

[1] Fan J., Zamani R., Fabrega C., Shavel A.,Flox C., M. Ibá˜nez M., Andreu T., L´opez A.M, Arbiol J., Morante J. R., Cabot A., Solution-growth and optoelectronic performance of ZnO: Cl/TiO_2 and ZnO: Cl/Zn_xTiO_y/TiO_2 core–shell nanowires with tunable shell thickness, *J. Phys. D: Appl. Phys*. **2012**, 45, 415301.

[2] Hernandez S., Cauda V., Chiodoni A., Dallorto S., Sacco A., Hidalgo D., Celasco E., Pirri C.F., Optimization of 1D ZnO@TiO2 Core-shell Nanostructures for Enhanced Photo-electrochemical Water Splitting under Solar Light Illumination, ACS Appl. Mater. Interfaces **2014**, 6, 12153–12167
